# Supplementary material for: All-at-once RNA folding with 3D motif prediction framed by evolutionary information
Source: Nat Methods. 2025 Oct 3;22(10):2094–106. doi: 10.1038/s41592-025-02833-w (PMC12510878; doi:10.1038/s41592-025-02833-w)
Supplement: Supplementary file 3 — Source code to run CaCoFold-R3D. Data and results. [file 41592_2025_2833_MOESM3_ESM.gz › supplemental_material/results/CaCoFold-R3D/CaCoFold-R3D_Rfam_vshuffle/RF02107_DLEU2_3.R2R.sto.pdf]

RF02107_DLEU2_3

5′-A A A G Y A ● A A U U U A C A A A ○ ○ ○ C A Y U U Y R U U C C U U G Y A G U A C A Y C U U U C A A A A G A C A U C U U C A U C A A G Y A G R U A A G A ●
